# Electron Paramagnetic Resonance Study of PbSe, PbTe, and PbTe:In Semiconductors Obtained by the Pulsed Laser Deposition Method

**DOI:** 10.3390/molecules27144381

**Published:** 2022-07-08

**Authors:** Aleksandra Wędrychowicz, Bogumił Cieniek, Ireneusz Stefaniuk, Ihor Virt, Romana Śliwa

**Affiliations:** 1 Doctoral School of Engineering and Technical Sciences, Rzeszow University of Technology, Powstańców Warszawy 12, 35-959 Rzeszów, Poland; 2 Institute of Materials Engineering, College of Natural Sciences, University of Rzeszow, Pigonia 1, 35-310 Rzeszow, Poland; bcieniek@ur.edu.pl (B.C.); istef@ur.edu.pl (I.S.); 3 Institute of Physics, College of Natural Sciences, University of Rzeszow, Pigonia 1, 35-310 Rzeszow, Poland; ivirt@ur.edu.pl; 4 Faculty of Mechanical Engineering and Aeronautics, Rzeszow University of Technology, Powstańców Warszawy 12, 35-959 Rzeszów, Poland; rsliwa@prz.edu.pl

**Keywords:** EPR, PbTe, PbSe, DMS, paramagnetic species

## Abstract

The magnetic properties of lead selenide (PbSe) and indium-doped lead telluride (PbTe:In) composites have been studied by using the electron paramagnetic resonance (EPR) technique. The samples were obtained by using the pulsed laser deposition method (PLD). Temperature dependences of the EPR spectra were obtained. The analysis of the temperature dependencies of the integral intensity of the EPR spectra was performed using the Curie–Weiss law. In these materials, the paramagnetic centers of Pb^1+^ and Pb^3+^ ions were identified. The results are discussed.

## 1. Introduction

The great interest in ferromagnetic semiconductors with a wide band gap [1,2] is related to the possibilities of their various applications, e.g., in spintronics and so-called translucent electronics [3,4,5]. The longitudinal optical phonons are the principal scattering mechanism in p-type PbTe. On the contrary, the scattering caused by transverse optical mechanisms is the weakest [6]. The density approximation results in an energy gap, the experimental value of which is greater than in most materials. The fitting of the experimental range was proposed using the Slater–Koster fitting method. Only the addition of a compression component to the narrowed Hamiltonian definition must result in significant modifications in the band structure [7]. The main difficulty in the production of materials with low thermal conductivity is the problem with the effective scattering of phonons in the entire frequency spectrum. Some calculations show that the passage of PbTe to a ferroelectric phase transition can provide a balanced solution to this problem [8]. In recent years, the topic of functional ferromagnetic semiconductors has aroused great interest. The ferromagnetic reaction usually takes place above room temperature in thin layers of semiconductors and oxides doped with small amounts of magnetic compounds [9,10,11]. EPR studies have proven that Cr^3+^ ions describe the n-type conductivity, as well as the magnetic properties of the PbCrTe compound. The paramagnetic resonance and its correlation with the chromium concentration prove the existence of a Cr donor resonance in PbTe. There is a noticeable shift in the g factor, which is caused by the increased concentration of the carrier. It is the earliest manifestation of the existence of a large sp-d coupling between Cr^3+^ ions and conducting electrons [12]. All samples show the dependence of the Curie temperature (T_c_) on the carrier concentration p, and it has the form of a threshold [13]. The result of the displacement of factor g is that the magnetic moment Mn^2+^of the semiconductor diagrams PbTe and SnTe is observed for n- and p-type crystals. The observation of this effect makes it possible to determine the carrier for holes and electrons in PbTe and light and heavy holes in SnTe [14]. Although PbTe is a reference thermoelectric material, its applications are still being investigated by attempts to change its properties [15]. There are models of thermoelectric transport mechanisms in the L and ƩPbTe valleys, with particular emphasis on thermally induced shifts. Semiconductors with magnetic properties are an interesting research object due to their spintronic properties and the interaction of spin–spin exchanges between localized magnetic moments and band electrons [16]. PbTe with an energy gap of 0.29 eV at a temperature of 300 K [17] are used for infrared detectors and solar cells [18,19,20]. Based on Bi_2_(Te, Se)_3_, Ag_2_Te, PbTe, and SnSe, functional fibers can be obtained as new flexible materials for thermoelectric devices [21,22]. Furthermore, there are potential applications of thermoelectric fibers or devices for electricity generation [23]. Due to the metallic conductivity of composites, the skin effect limits the penetration depth of microwave radiation to approximately10 µm, resulting in an asymmetrical (dissonant) shape of the resonance lines [24]. The most interesting effects related to the influence of electronic properties of semi-magnetic semiconductors on their magnetic behavior are observed in the IV–VI groups of semiconductors, such as PbSe or PbTe [25,26,27,28,29]. Interesting ferromagnetic properties have been observed in many diluted magnetic semiconductors (DMS), e.g., ZnTe + Mn [30] and CdTe + Cr [31]. In addition, interesting ferromagnetic properties have been studied in works [32,33,34,35] for the doping of the Cu ion in ZnO. Although Cu, CuO, and Cu_2_O are not ferromagnetic, typical ferromagnetic interactions of DMS are observed. Depending on the oxidation state of the copper ion, paramagnetic Cu^2+^, and diamagnetic Cu^+^, we can “switch” the magnetic properties by interactions with the defects. The introduction of hydrogen (H^+^) into the (Zn, Cu)O layer results in the appearance of ferromagnetic properties because of the interaction with the defects. Curie temperatures of 42.5 K (spin-only) and 106.1K (spin–orbit coupling), respectively, were observed, depending on the type of defect and associated interactions [35]. A high Curie temperature (T_C_ ≈ 320 K) and band gap opening are two key challenges (MnSe_2_) for the integration of photoconductivity into two-dimensional (2D) magnets [36]. The primary subject of this paper is a review of the magnetic properties of the PbSe, PbTe, and PbTe:In layers studied by the EPR method, learning about these magnetic properties in combination with electrical measurements can be helpful in building thermoelectric devices.

## 2. Results and Discussion

### EPR Measurements

**Figure 1:**
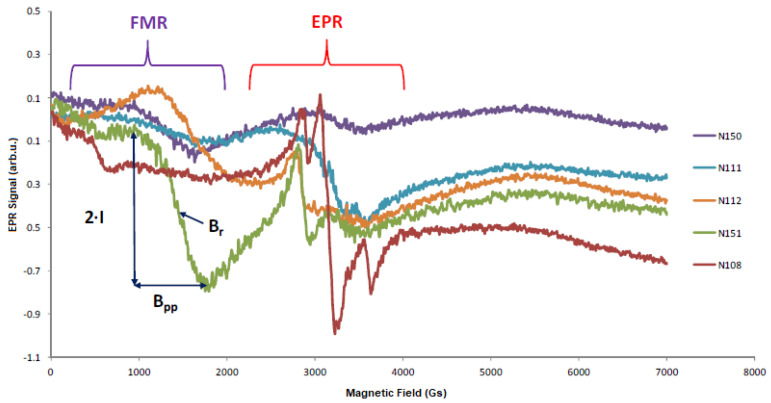
EPR measurements of PbTe (N111), PbTe (N112), PbTe (N150), PbTe:In (N151), and PbSe (N108) at room temperature. FMR-line area for ferromagnetic resonance, EPR- line area for paramagnetic resonance, B_pp_-peak-to-peak width of the resonance line, I-resonance line intensity, and B_r_-resonance field.

X-band EPR spectra were measured for all samples, Figure 1 shows the spectra at room temperature (RT).

All spectra are characterized by an EPR line in the vicinity of 300 mT. For samples N151, N112, and N150, a wide line was observed in a low field of about 150 mT. However, in the case of the EPR spectrum for PbSe, we observed a typical Pb^2+^ ion line with a visible hyperfine structure in the vicinity of 300 mT. The configuration of the outer electron shell of Pb is 6s^2^6p^2^ and, thus, S = 1, L = 1, and J = L-S ≠0. Therefore, the conditions enabling the prevalence of polarization para-magnetism of an atomic electron shell over its precession diamagnetism are fulfilled. On the other hand, in the PbTe interstitial, the Pb atom will be surrounded by alternatively positioned ions of opposite signs, Pb^2+^and Te^2-^, forming a PbTe lattice of the NaCl type [1]. As a result, the outer electron shell of interstitial Pb will experience a strong attraction to Pb^2+^ ions of the metal sublattice and a strong repulsion from Te^2-^of the chalcogen one. Thus, the interstitial Pb in the PbTe lattice can be a strong Van Vleck-type paramagnetic center. The Pb^2+^ ion has the electron configuration 6s^2^ and therefore has no electron magnetic moment and no observed EPR spectra. Lead ions, Pb^+^ and Pb^3^^+^, have electronic configurations 6s^2^p^1^ and 6s^1^, respectively. In the EPR spectra of both ions, a hyperfine structure induced by the interaction of an unpaired electron with the magnetic moment of the 207Pb nucleus (spin l = 1/2, natural content 21.1%) should be observed. The natural occurrence of even Pb isotopes without a nuclear spin (I = 0) is 78.9%. EPR spectra for Pb ions have been observed in many works, (see, e.g., [37,38,39,40,41]). They are successfully described using a rhombic-symmetry spin Hamiltonian (1) with an electron spin S = 1/2 and nuclear spins I = 0 or ½ of the form (in the usual notation).

(1) $$H_{s}=g\beta \mathrm{BS}\;+\;\mathrm{AIS}$$

where: g—spectroscopic splitting factor, β-Bohr magneton constant, B-magnetic field, A-hyperfine structure tensor, I-nucleus spin, and S-total spin.

**Figure 2:**
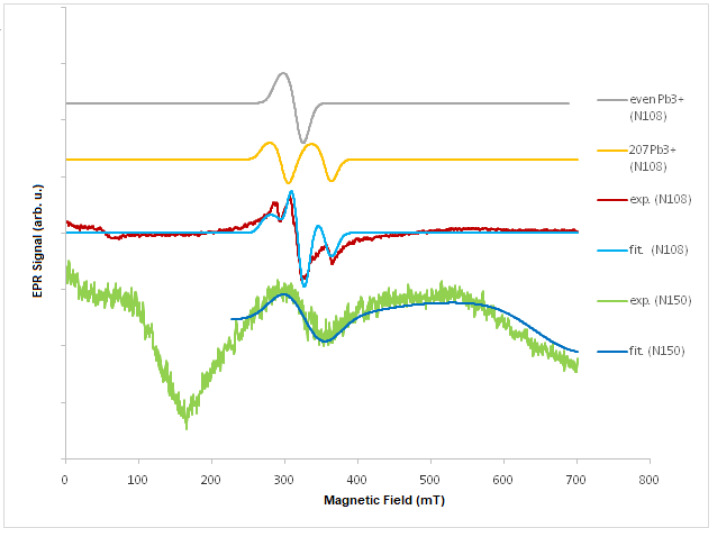
EPR spectra at room temperature for PbTe (N150) and PbSe (N108) experimental (exp.) and fitted (fit), as well as an example of simulated EPR spectra for 207Pb^3+^ ion and even Pb isotopes (206Pb, 208Pb, and 210Pb).

Based on this relationship, the factor g_eff_ values were determined (see Table 1), while, for PbSe, the hyperfine structure constant A. Moreover, we performed fitting and simulation using MATLAB software with an Easyspin toolbox for both Pb^3+^ and Pb^1+^ ions at room temperature. The obtained results are shown in Figure 2. The parameters of the spine Hamiltonian (Equation (1)) were determined. For the Pb^1+^ ion, g = (1.000, 1.002, 1.504) and A = (5.20, 3.62, 4.58) MHz (N108); for the Pb^3+^ ion, g = (2.105, 2.106, 2.105), A = (1584.0, 1579.7, 1757.0) MHz (N150), and a g = (2055, 2006, 2216) and A = (1634.0, 1673.6, 1754.0) MHz (N150). In the literature (e.g., [41]), for Pb^+^, g_eff_ = 1.12, and, for Pb^3+^, g_eff_ ≈ 2. We can assume that we mainly observed the EPR spectrum from Pb^3+^ ions and, for 3 samples, additional magnetic interactions.

**Figure 3:**
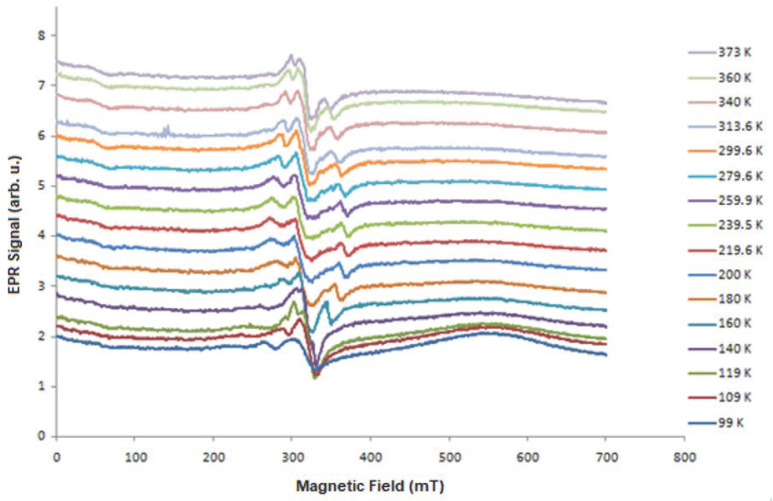
Temperature dependence of the PbSe EPR signal (N108).

**Figure 4:**
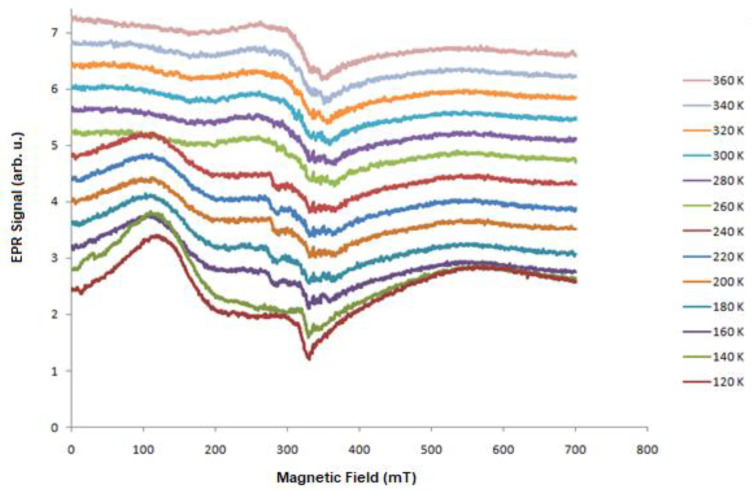
Temperature dependence of the PbTe EPR signal (N111).

**Figure 5:**
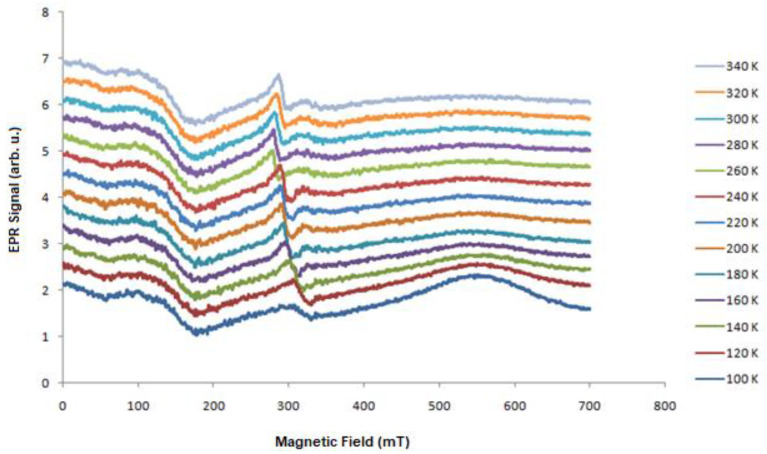
Temperature dependence of the EPR signal of PbTe:In (N151).

To characterize the samples from a magnetic point of view, we made dependencies of the EPR spectra as a function of the temperature. Figure 1, Figure 3, Figure 4 and Figure 5 present the EPR spectra.

**Figure 6:**
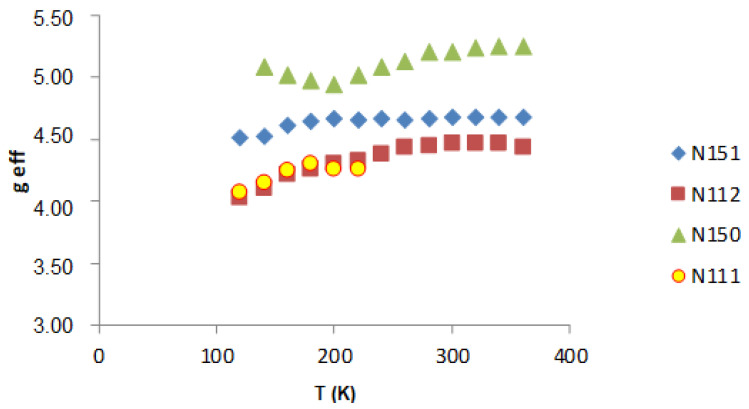
g_eff_ as a function of the temperature for the “ferromagnetic” line (g_eff_ ≈ 5).

**Figure 7:**
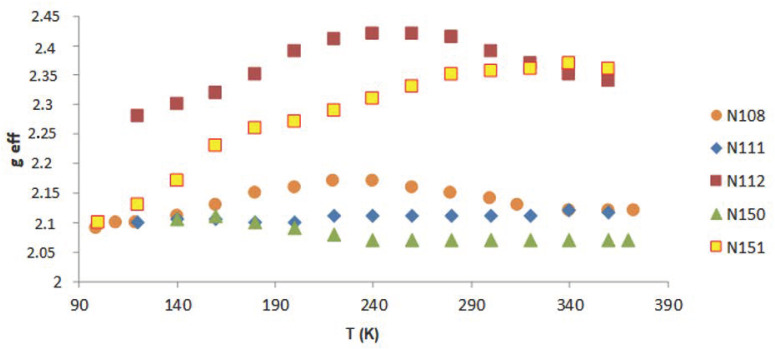
g_eff_ as a function of the temperature for Pb^3+^ (g_eff_ ≈ 2).

**Figure 8:**
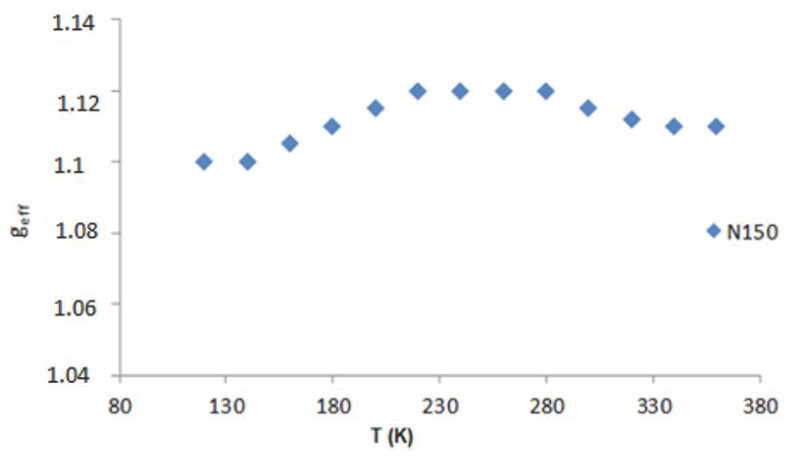
g_eff_ as a function of the temperature for Pb^1+^ (g_eff_ ≈ 1.1).

On the basis of the performed EPR measurements, the line parameters were determined, peak-to-peak width (B_pp_), line intensity (I), and resonant field (B_r_). From the dependence (1), the value of the factor g_eff_ was determined. Figure 6, Figure 7 and Figure 8 show the dependence of g_eff_ as a function of temperature for the “ferromagnetic” line (g_eff_ ≈ 5), the Pb^3+^ (g_eff_ ≈ 2), and Pb^1+^ (g_eff_ ≈ 1.1). For the EPR line with g_eff_ ≈ 1.1, we observe the overlapping of signals from the Pb^1+^ ion and various defects related to oxygen ions [42]. At lower temperatures, there is a clear separation of these lines, and for the Pb^1+^ ion, we can observe a clear and narrow line (e.g., sample N150), while, in the other samples, this line is much weaker.

The greatest differences are observed for the “ferromagnetic” line, whereas the g_eff_ value for the Pb^3+^ ion is constant in this temperature range.

An analysis of the changes in the EPR spectrum as a function of temperature on the total intensity of the EPR spectrum was performed. The total intensity of the EPR spectrum is determined on the basic line parameters according to the relation:

(2) $$I_{\mathrm{EPR}}=\;I{\left(B_{\mathrm{pp}}\right)}^{2}$$

**Figure 9:**
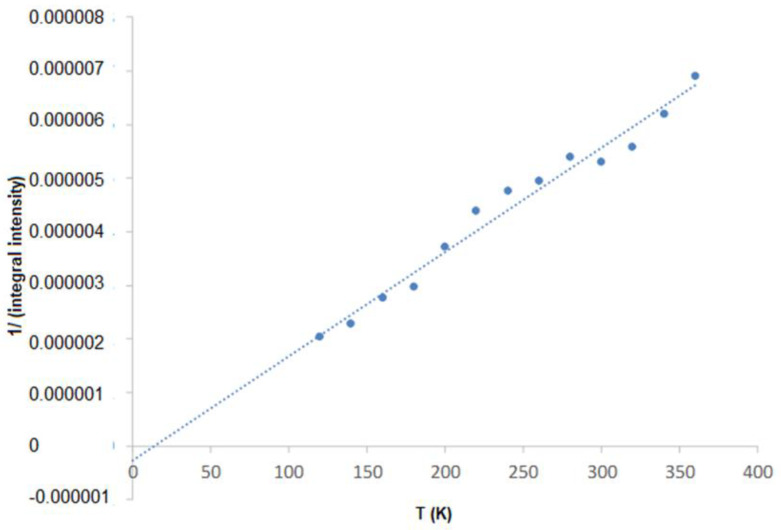
Temperature dependence of the 1/(integral intensity) of PbTe (N112).

We use the Curie–Weiss law to analyze the temperature dependence of the integral intensity, which is directly proportional to the magnetic susceptibility χ. A linear increase of χ^−1^(T) at higher temperatures can be fitted to the Curie–Weiss law:

(3) $${\left(\chi -\chi_{0}\right)}^{-1}\left(T\right)=\frac{T}{C}-\frac{T_{C}}{C}$$

where C is the Curie constant, Tc is the paramagnetic Curie temperature, and $\chi_{0}$ is a temperature-independent term to account for the diamagnetic host and any Pauli Paramagnetism contribution. An example of the relationship ${\left(\chi -\chi_{0}\right)}^{-1}\left(T\right)$ for the N112 sample is shown in Figure 9. The calculated values of the Curie temperature and the Curie constant for all samples are presented in Table 1. The lines are linear extrapolations illustrating the ferromagnetic (positive) Curie-Weiss temperatures.

When analyzing the obtained results, depending on the parameters of the obtained layers, we notice that ferromagnetic interactions appeared in only four samples. For sample N112, we observed a weak “ferromagnetic” line, and it was in correlation with the low Curie temperature. For sample N111, we observed the appearance of lines from ferromagnetic interactions at a temperature of about 240 K.

The highest ferromagnetic interactions were observed for PbTe (N111) and indium-doped PbTe (N151). In addition, the substrate temperature for the range of 200–250 K has a large influence, the PbTe layers have ferromagnetic properties, and, for a lower temperature, we do not observe ferromagnetic properties, while, at 300 K, these properties are still visible but weaker, and the obtained Curie temperature is lower. The appearance of the low-field line (LFMA) together with the “ferromagnetic” line is interesting; especially, it is visible in Figure 4 (N111). Different interpretations and explanations have been presented to try and explain the appearance of the LFMA signal at B = 0 in a wide variety of materials. For magnets and ferrites, the LFMA signal is associated with the onset of the ordered phase and provides a sensitive detector of magnetic ordering [43,44]. For soft magnetic materials, the LFMA signal is due to low-field spin magnetization processes [45]. In our case, we connected the appearance of the LFMA signal with the occurrence of ferromagnetic properties.

## 3. Materials and Methods

The experimental setup of the pulsed laser deposition method (PLD) used to deposit the PbSe and PbTe layers has previously been extensively described [46] and is only briefly described here. It uses a Q-switched Nd^3+^: KGd(WO_4_)_2_ laser (λ = 1067 nm, pulse duration τ = 20 ns, 6–8 J/cm^2^ fluence, and 0.3 Hz repetition rate) to ablate the polycrystalline targets in a quartz steel chamber. The final pressure in the deposition chamber was in the low 10^−6^ Pa. The layers were deposited under residual vacuum. For various samples, the temperature of the substrate (T_s_) was changed, as well as the layer deposition time, related to the number of pulses and, hence, their thickness. The layers were prepared on quartz for EPR measurements (Table 2) and Al_2_O_3_ for electrical measurements (Table 3).

EPR measurements were performed in X-band (~9.5 GHz) using a Bruker EleXSYS-E580 spectrometer (Billerica, MA, USA and Karlsruhe, Germany) equipped with a Bruker liquid Ngas flow cryostat with the 41131 VT digital controller (Bruker Analytische Messtechnik, Rheinstetten, Germany) within the temperature range 100–400 K.

Measurements of the electrical parameters of the semiconductor layers were performed on the developed automated installation developed according to the classical method. After applying a voltage to the sample (10 V), the current flowing is measured. During the measurement, the sample (layer) was placed in a standard copper-based holder with four measuring probes and a built-in reference resistor for current measurement with a digital microvoltmeter. The handle is attached through a detachable joint in the middle of a glass cylinder, in which a precise temperature sensor is mounted on a thermocouple connection. The production of reliable ohmic contacts that do not damage the layer and meet all the requirements was carried out by pre-brazing indium traces on the surface of the Al_2_O_3_ substrate prior to the production of the layer.

**Figure 10:**
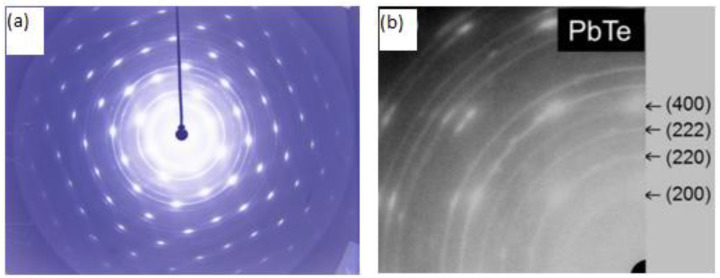
THEED patterns of (**a**) PbTe:In and (**b**) PbTe. The most pronounced Miller indices (hkl) are indicated.

**Figure 11:**
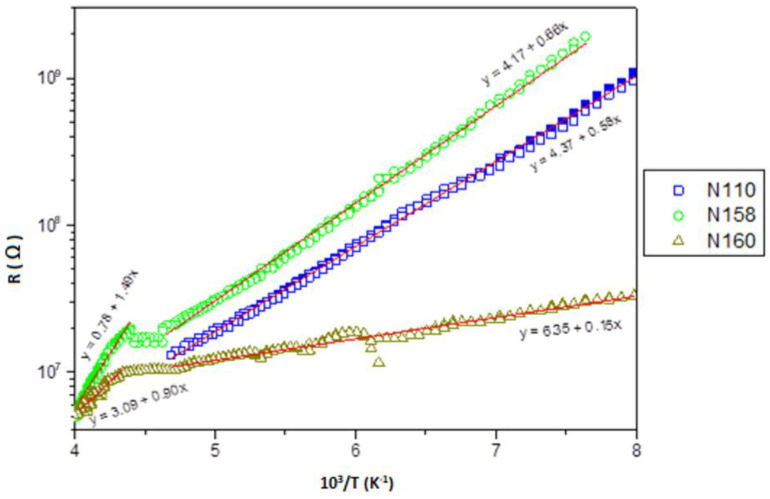
Electrical resistance as a function of the temperature of PbTe (N110), PbTe:In (N158), and PbSe (N160).

The structural quality of the respective films was investigated by transmission high-energy electron diffraction (THEED) for the samples grown on KCl (001) substrates. We used an EMR-100 electron diffractometer with acceleration voltages of 60–80 kV. For a better explanation of the good crystalline quality of the samples, in Figure 10, a summary of THEED images (a) for the PbTe:In layer studied and (b) PbTe published in Figure 10a) in [33]. All samples in Table 2 are of similar quality, as can be seen in the THEED image in Figure 10a. However, the same layers with different thicknesses are described in [33], and the parameters of the elemental cell are determined for them, along with the assigned Muller indexes. For example, the PbTe layer (Figure 10b) from Figure 11 [33].

### Electrical Measurements

The properties of the established contacts were controlled by analyzing the volt-ampere characteristics of the samples. The measurement temperature range was 77–500 K (sample temperature change).

Measurement soft electrical resistance as a function of temperature are shown in Figure 11. The type (carrier type) of conductivity is defined by the sign of the thermoelectromotive force (t_ers_). The electrical characteristics of the layers are presented in the following coordinates: resistivity to inverse temperature (ρ 10^3^/T).

The dependence of the resistance as a function of the temperature is linear over a wide range for all samples, and we see only differences in the slope of this line. On the other hand, for both samples, PbSe and PbTe:In at about 220 K, we observe a change in the slope of the straight line. In all samples, two drops in activation energy, depending on the resistivity temperature, are related to self-conductivity (at relatively high temperatures) and to doped conductivity (at temperatures below 200 K).

In paper [33], THEED images with high reorientation of crystallites were observed, practically only Debye-Scherrer rings and weakly visible reflections were visible. On the other hand, the RHEED images presented in Figure 10 are characterized by very good reflections with weakly visible Debye-Scherrer rings.

## 4. Conclusions

Electrical measurements were carried out to indicate that the obtained materials are of a semiconductor nature. All samples observed two activation energy slopes on the temperature dependence of resistivity: related to self-conductivity at relatively high temperatures and to a doped conductivity at temperatures below 200 K.

Identification of the paramagnetic centers present in the tested materials was carried out using EPR measurements. It was determined that the EPR spectrum comes from the Pb^1+^ ions in the sample (it is most visible in sample N150) and from Pb^3+^. The g_eff_ values were found, which agree well with the data reported in the literature. In addition, we observed additional defects, i.e., vacancies and interstitial ions, which cause a widening of the line for g_eff_ ≈ 2 and g_eff_ ≈ 1.1. The natural oxidation state of the Pb ion is Pb^2+^, while our measurements indicate the presence of both Pb^3+^ (g_eff_ ≈ 2) and Pb^1+^ (g_eff_ ≈ 1.1) ions. The EPR line derived from Pb^1+^ ions was weaker, due to the large broadening of the line and, thus, lower intensity. The observation of these two ions is related to the charge compensation in all samples. The Pb^2+^ ions are probably responsible for the ferromagnetic interactions (g_eff_ ≈ 5). Pb^1+^ ions are observed at low temperatures in all samples.

A hyperfine structure for PbSe was observed. The determined temperature dependence was almost linear, while the constant value A = 1740 MHz (at 109 K) changed as a function of the temperature. The highest value of A = 2533 MHz was taken at 240 K and A = 1392 MHz at 373 K.

Ferromagnetic interactions were observed in samples N111, N112, N150, and N151. The greatest effect was observed in PbTe (N111). The temperature of the substrate has a large impact on the ferromagnetic properties; the lower it is, the better the ferromagnetic properties, while the admixture of indium gives similar properties at a higher temperature of the substrate. The temperature range of 200–240 K was also defined, in which it was possible to obtain DMS.

An interesting effect was observed for the temperature around 200–240 K. There is a change in the slope of the temperature dependencies g_eff_, especially for the ferromagnetic line g_eff_ ≈ 5 (Figure 6), and it is in correlation with the electrical measurements (Figure 11). A likely source of ferromagnetism is localized electrons that, under increasing temperature, move into the conduction band and undergo delocalization. This is particularly clear for sample N111, for which the Curie temperature is near this range, and for this sample, we observe a decay of ferromagnetic properties above 240 K. Since the Curie temperatures of the other samples are low, this effect is not observed, and we only see changes in the value of the spin–orbit coupling for the g-factor example. Further research of these materials is planned to improve the ferromagnetic properties and obtain the highest Curie temperature.

## Figures and Tables

**Table 1:** Summary of the Curie temperature with constant and g_eff_ parameters from EPR measurements at room temperature.

	Magnetic Properties		g_eff_ (RT)		
Sample	T_c_(K)	C	“Ferromagnetic” Line (g_eff_ ≈ 5)	Pb^3+^ (g_eff_ ≈ 2)	Pb^1+^ (g_eff_ ≈ 1.1)
N108	-	-		2.14	
N150	75.83	3.31 × 10^7^	5.20	2.07	1.115
N151	132.99	1.94 × 10^6^	4.67	2.36	
N112	5.31	1.79 × 10^8^	4.46	2.39	
N111	174.21	1.59 × 10^10^	-	2.11	

**Table 2:** Layer growth parameters deposited on a quartz substrate for EPR measurements.

	N111	N112	N150	N151	N108
	PbTe	PbTe	PbTe	PbTe:In	PbSe
**T_s_(°C)**	120	200	300	250	220
**Time (min)**	20	25	25	30	25
**Number** **of** **pulses**	400	500	500	600	500

**Table 3:** Layer growth parameters deposited on the Al_2_O_3_ substrate for electrical measurements.

	N110	N158	N160
	PbTe	PbTe:In	PbSe
**T_s_(°C)**	200	200	200
**Time (min)**	25	45	45
**Number** **of** **pulses**	300	550	550

## Data Availability

The data presented in this study are available on request from the corresponding author.

## Sample Availability

Samples of the compounds are not available from the authors.
